# Researches into the Real Constitution of the Atmosphere

**Published:** 1842-03

**Authors:** 


*Researches into the real constitution of the Atmosphere.* By MM. Dumas and Boussaingault.—It is generally admitted that the air is composed of a mixture of oxygen and nitrogen, and its invariableness is explained by supposing that the green parts of plants under the influence of solar light decompose all the carbonic acid developed in the respiration of animals, and the putrefaction of organized bodies. Some, however, regard the air as being not a mixture, but a chemical compound of 20 of oxygen and 80 of nitrogen (Prout, Doberein-er, Thomson, &c). Others, and these the majority, consider it as a mixture of 21 of oxygen and 79 of nitrogen; and, lastly, in the opinion of some (Dalton, Babinet), the composition of the air varies according to the height in the atmosphere.

The plan employed by the authors in submitting these questions to a fresh examination is distinguished from others in that they estimate the weight instead of the measure of the gases, and thus analyze the air by weighing successively the oxygen and the nitrogen which it contains. We cannot follow them into all the details of their experiments, which, by successive corrections, were rendered more and more exact: we can only point out the results.

They fix the density of oxygen at 1.1057, and that of nitrogen at 0.972; numbers a little different from those given by other chemists. They demonstrate that the relation of the volume of the oxygen to that of the nitrogen in the air is not expressed by simple numbers; and that the air cannot be regarded as a chemical compound of 20 volumes of oxygen and 80 of nitrogen. They admit, as *a sufficient approximation*, that the atmosphere is composed, by volume, of 20.8 of oxygen, and 79.2 of nitrogen. They presume that the mixture is uniform in all times, in every latitude, and at every height. "If the atmospheric air," they add, "is a reservoir of oxygen for the use of animals, and a reservoir of carbonic acid for the use of plants, it is so considerable a store that the consumption, supposing it not to be compensated, would remain almost insensible after a long series of years." They have calculated that supposing each man to consume a kilogramme of oxygen per day, and that the oxygen disengaged by plants did no more than compensate for the other causes of its absorption, the whole human race, and three times their number, would not consume, in a century, the eight-thousandth part of the oxygen which nature has placed in the respirable air.—*Lond. Med. Gaz.*, Oct. 15, 1841, from *L'Examinateur Medical*, Aout 20, 1841.

